# Price discovery effects of Federal Milk Marketing Order modernization: Evidence from cheese survey and class I mover revisions

**DOI:** 10.3168/jdsc.2026-1063

**Published:** 2026-06-04

**Authors:** Avery J. Pound, Aidan T. Ainslie, Christopher A. Wolf

**Affiliations:** Dyson School of Applied Economics and Management, Cornell University, Ithaca, NY 14853

## Abstract

Federal Milk Marketing Orders translate surveyed wholesale dairy commodity prices into minimum class prices and a pooled uniform price from which farm milk prices are constructed. Following a 2023–2024 hearing, the USDA's 2025 final rule introduced 5 categories of change to milk pricing. We focus on 2 changes with implications for price behavior and incentives: the cheese price survey eliminated 500-lb Cheddar barrels, using only 40-lb blocks, and the class I skim mover reverted to the *higher-of* the advanced class III or IV skim (with an extended shelf life adjuster). We find evidence that removing barrels, in isolation, is likely to modestly increase the price level in the cheese survey and derived protein series relative to combined blocks and barrels. We also conclude that returning to *higher-of* is likely to increase the average class I skim milk price mover relative to *average-plus* price rule. Collectively, the changes sharpen price signals for farm milk while supporting conditions associated with orderly marketing.

## Highlights

• 2025 FMMO reforms revised the class I mover and dropped barrels from cheese surveys.
• *Block-only* cheese pricing raises protein values and farm milk prices modestly.
• *Higher-of* class I pricing increases average skim prices by about $0.25/cwt.
• Reforms strengthen price signals and support class I as the highest-valued use.

## Graphical Abstract

**Figure:**
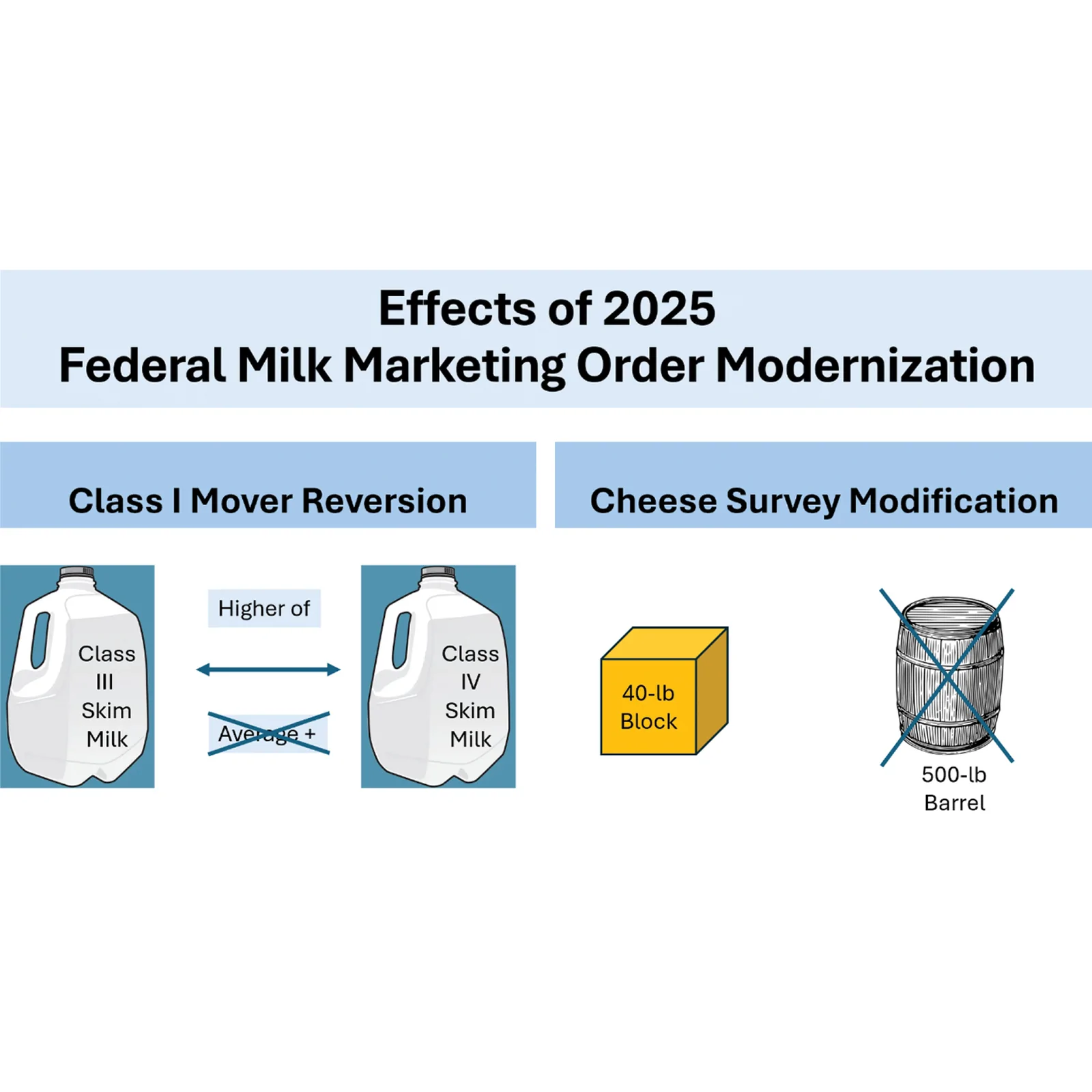
**Summary:** In 2025, the United States Department of Agriculture (USDA) implemented Federal Milk Marketing Order (FMMO) reforms that revised how class prices are calculated. We evaluated the price effects of 2 changes: removal of 500-lb Cheddar barrels from the cheese price survey, and reversion of the class I skim mover to the *higher-of* the advanced class III or IV skim. We found that eliminating barrel prices modestly raises the cheese price survey and protein values, particularly during lower-margin periods. Returning to the *higher-of* class I skim mover increases the average class I skim price by roughly $0.25/hundredweight (cwt) relative to the *average-plus* rule, while restoring asymmetric price risk from fluid processors to producers. Together, these reforms strengthen manufacturing price signals and reinforce class I's role as the highest-valued use, supporting orderly marketing.

Federal Milk Marketing Orders (**FMMO**) have affected prices paid to US dairy farmers since the 1930s. The system was designed to promote orderly marketing of fluid milk, balance bargaining power between producers and processors, and ensure adequate supplies of grade A milk in geographically defined markets. These objectives are implemented primarily through classified pricing, which assigns milk to end-use classes, and pooling, which redistributes revenue across producers within an order irrespective of how individual milk shipments are used.

Changes in milk production, processing technologies, product mix, and market volatility have periodically prompted revisions to FMMO pricing rules. The most recent reform efforts followed a 49-d USDA hearing held during 2023–2024, which considered more than 20 proposals with extensive testimony from producers, cooperatives, processors, and other stakeholders. This process culminated in a final USDA decision issued in January 2025, and approved by producer referenda in each of the 11 federal orders. The adopted amendments represent the most comprehensive revision to national FMMO pricing provisions since the late 2000s (Nicholson et al., 2025).

The final rule included 5 categories of changes affecting minimum class price formulas: (1) updates to class III and class IV manufacturing (make) allowances and the butterfat recovery factor; (2) revisions to surveyed dairy products used in pricing formulas, including removal of 500-lb Cheddar barrels from the cheese price survey; (3) a change to the base class I skim milk pricing formula, returning to the *higher-of* the advanced class III or class IV skim milk prices; (4) updates to class I location differentials; and (5) updates to standardized skim milk composition factors. Most provisions became effective June 1, 2025, with composition factor changes implemented on December 1, 2025, to allow adjustment in risk-management positions.

Although all 5 changes affect farm milk pricing, this research focuses on 2 reforms that directly alter benchmark price discovery within the FMMO system: the elimination of barrel Cheddar prices from the cheese survey used in class III pricing, and the return to the *higher-of* class I skim milk pricing rule. The price discovery effects of these 2 changes are examined using descriptive statistics and frequency analysis. We use FMMO data on commodity and class price series from 2014 to 2024 to assess the impacts of these changes (USDA AMS 2025a,b). Specifically, we compare benchmark cheese prices under alternative survey definitions and examine how the *higher-of* versus *average-plus* class I skim milk mover affects average prices, variability, and the probability that class I prices exceed alternative benchmarks. These changes modify how market prices are incorporated into regulated formulas. By focusing on these 2 pricing-rule changes, the analysis highlights how relatively narrow formula revisions can materially influence regulated price discovery and risk exposure in US milk markets, independent of downstream revenue or welfare effects. This article makes a contribution by isolating the mechanical price discovery effects of 2 core FMMO pricing reforms using observed historical data.

Minimum farm class milk prices are derived from product prices. Wholesale dairy product prices for 4 manufactured products (cheese, dry whey, butter, and nonfat dry milk) are surveyed weekly to determine monthly averages. Milk component values are determined using these wholesale prices as well as allowances for manufacturing costs and yields.

The first change considered modified which products were surveyed to determine wholesale cheese prices. Under the previous procedures wholesale Cheddar prices were surveyed using a weighted average of 40-lb (18.14-kg) blocks and 500-lb (226.8-kg) barrels. Block cheese is used for many different products in its natural form, whereas 500-lb barrels tend to be used for processed cheese products. The FMMO reform drops barrel cheese prices from the calculation and derives the wholesale cheese price solely from 40-lb (18.1-kg) blocks of Cheddar. The cheese survey change reflects USDA's determination that 40-lb block and 500-lb barrel Cheddar cheeses had become distinct products serving different markets, with increasingly volatile price spreads. By removing barrels from the survey, the revised rule alters the representation of cheese market conditions used to calculate class III prices and associated protein component values.

Table 1 displays summary statistics for wholesale cheese prices over the period from January 2014 through December 2024. The column labeled *including-barrels* is what actually occurred as a weighted average of the block and barrel prices. The column labeled *block-only* is a counter-factual price that includes only the block Cheddar price. Over the 11-yr period, Cheddar blocks averaged $1.81 lb, whereas including barrels resulted in a weighted cheese price of $1.788/lb for a difference of $0.022/lb. As COVID-19 was very disruptive to dairy markets, we also examine just the period following the major effects of the pandemic. Considering only 2021–2024, the average cheese price was $0.015/lb higher when barrels were not included in the calculation. The price benefit to dropping barrels is negatively correlated with farm profit margins (i.e., barrel discounts tend to widen in weak markets) so that it increases farm prices more in periods of lower profit.

**Table 1:** Cheese prices: monthly summary statistics of Cheddar prices^1^

Time period	Statistic	*Including-barrels* cheese price, $/lb cheese	*Block-only* cheese price, $/lb cheese
2014–2024	Average	1.788	1.810
	SD	0.272	0.272
	Minimum	1.299	1.305
	Maximum	2.587	2.698
2021–2024	Average	1.853	1.868
	SD	0.239	0.223
	Minimum	1.487	1.461
	Maximum	2.416	2.380

1 Data source: USDA Agricultural Marketing Service (2025a). Announcement of class and component prices.

The wholesale Cheddar cheese price is used to derive the protein component value. The change to using only block prices manifests in farmer pay as a change in protein price paid. Everything else being equal, specifically keeping make allowances constant at their previous level to isolate the effect of the cheese survey change, the change to block only cheese price increases protein price by $0.05–$0.07/lb depending on the time period considered. To convert to a hundredweight (**cwt**) farm price, multiply by the pounds of protein in a hundred pounds of milk, which averages a bit more than 3%. Thus, the 7 cents per pound of protein results in an increase of about 22 cents per cwt of milk paid to the farmer.

The second price modification examined determines how class I skim milk prices are set. Class I, fluid product, minimum prices are constructed from a base that considers both class III (cheese and dry whey) as well as class IV (butter and nonfat dry milk) prices. To facilitate retail pricing, regulated prices for class I products are set before the start of the month. Weighted average prices from the first 2 wk of each month are used to calculate the “advanced prices” for the following month. Advanced prices are announced by the 23rd of the month for the following month, which is weeks earlier than the other final class prices. The *higher-of* method, defined as the maximum of either the advanced class III or IV skim milk price that month, was used to calculate the skim milk price of class I milk from January 2000 until May 2019 when the 2018 Farm Bill (Agriculture Improvement Act of 2018; US Congress, 2018) mandated that the FMMO class I skim milk price be calculated as the average of the monthly class III and class IV advanced pricing factors plus $0.74 per cwt (hereafter *average-plus*). When the *average-plus* method replaced the *higher-of* in May 2019, the $0.74/cwt differential was chosen because it equaled the average difference in the pricing method values from 2000 through 2017 (Newton, 2020). Under the *higher-of* method, class I processors could not be certain in advance whether the class III or IV skim milk price would set their input cost in a given month as these prices fluctuate. Wide divergences in class III and IV milk price along with the *average-plus* pricing rule sometimes generated months in which the class I price was not the highest priced class use. This situation contributed to disorderly marketing in terms of negative producer price differentials and incentive for some organizations to depool milk (Bozic and Wolf, 2022). The USDA final decision that resulted from the hearing process returned to the *higher-of* class III or IV skim milk price but also adopted a class I ESL adjustment.

Table 2 displays summary statistics for the 2 pricing rules as well as the difference between the monthly values from 2014 through 2024 as well as 2021 through 2014, which avoids the most severe market impacts of COVID-19. The *higher-of* pricing rule results in a larger average class I skim milk price than *average-plus* in both periods considered. Over the 2 time periods considered the *higher-of* would have generated $0.26–$0.27/cwt greater class I skim milk prices. The *higher-of* generated this larger average class I skim milk price because of relatively frequent large divergences between class III and IV price. Over the longer period, the *higher-of* pricing rule generated a wider range of values with a larger maximum and a smaller minimum.

**Table 2:** Summary statistics of advanced class I skim milk price^1^

Time period	Statistic	Using *average-plus* method, $/cwt	Using *higher-of* method, $/cwt
2014–2024	Average	9.71	9.98
	SD	2.91	3.30
	Minimum	5.57	5.31
	Maximum	17.80	20.07
2021–2024	Average	10.52	10.88
	SD	2.09	1.96
	Minimum	7.18	8.55
	Maximum	15.66	15.04

1 Data source: USDA Agricultural Marketing Service (2025b). Announcement of advanced prices and pricing factors.

The larger average class I skim milk price would indicate that the return to the *higher-of* will likely increase farm milk price but that is not always the case. Figure 1 displays the difference between the *higher-of* and *average-plus* class I skim milk price from 2014 through 2024. The values are positive when the *higher-of* would have returned a larger price and negative when the *average-plus* would have returned a larger price. *Average-plus* $0.74/cwt returns a higher class I skim milk price when the difference between the advanced class III and IV skim milk prices differ by less than $1.48/cwt in a given month. Thus, the negative values in Figure 1 represent class I skim milk price declines—losses from the farm price point-of-view—in that month using the *average-plus* pricing rule. The figure demonstrates the asymmetry in price differences using the 2 rules. The maximum advantage of the *average-plus* rule is capped at the value added—in this case $0.74/cwt (represented by the red line in Figure 1). Meanwhile, the potential benefit of the *higher-of* rule for class I skim price is that it can reach much larger values. COVID-19 was the extreme case of advanced class III and IV skim milk price divergence which was smoothed out by the *average-plus* rule.

**Figure 1:**
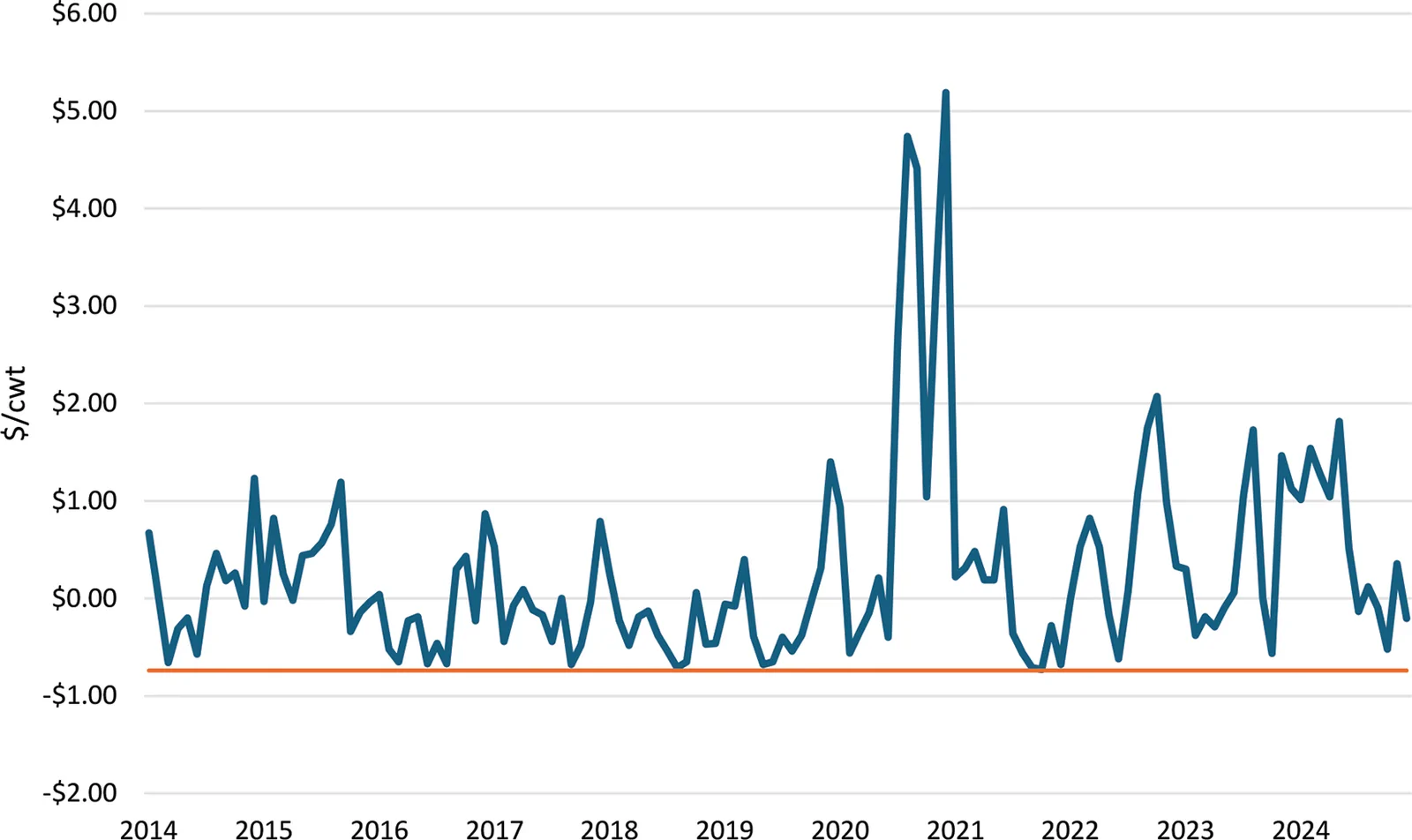
Difference Between *higher-of* and *average-plus* class I skim price. Data: author calculations using USDA Agricultural Marketing Service (2025b). Announcement of class and component prices. Note: the blue line is positive when *higher-of* was advantageous to class I skim milk price; the red line is the −$0.74/cwt floor *higher-of* advantage.

Figure 1 reveals that class III and IV prices were near each other often as the loss using the *higher-of* rather than the *average-plus* was frequently near the $0.74/cwt price floor difference. The *average-plus* results in a lower class I skim milk price than the *higher-of* in any month when the absolute difference between class III and class IV advanced skim milk prices differ by more than $1.48/cwt. The risk borne, in the sense of foregone revenues, by dairy farmers using the *average-plus* pricing model is larger than that borne, in the terms of input prices, by fluid processors. This asymmetry occurs because class III and IV advanced skim milk price can often diverge by large absolute values. The *higher-of* would have generated a higher class I skim milk price in 35% of months 2014–2024 and 50% of the months 2021–2024. Thus, whereas the *higher-of* generates larger prices in less months than the *average-plus*, the potential gain in farm milk price from the *higher-of* rule is not capped. In recent years, the difference between the class III and IV skim milk price often exceeded $1.48, reaching $11.86/cwt in December 2020.

By its nature, the *higher-of* pricing rule generates higher prices when there is a large change, either positive or negative, in class III or IV milk price relative to the other. The psychology of the 2 rules is important to understand dairy farmer preferences. Loss aversion refers to the concept that losses are more psychologically impactful than gains. Although the *average-plus* provides price upside in many months, the potential for large downside in terms of large foregone revenues under the *higher-of* rule means that many dairy farmers find it preferable. Since the class I skim milk price rule was changed in 2019, the divergence in class III and IV price was historically high until reform changed it back in June 2025. Couple that with the ability to anchor the price to the previous pricing rule and it becomes apparent why farmers preferred a change back to the *higher-of* rule.

To accommodate the longer-term contracting aspects of extended shelf-life (**ESL**) milk, the reform provided an adjustment to advanced class I skim milk prices for all ESL products equal to the average of the advanced class III and IV skim milk prices in a given month plus a 24-mo rolling average adjuster with a 12-mo lag. The ESL adjuster will be revenue neutral for farmers over a 36-mo period and serves as a benefit to the risk management of co-operatives and processors making ESL. It will be interesting to see how this change affects the incentive to produce ESL milk.

The 2025 modernization of FMMO included multiple changes, but 2 directly reshaped regulated price discovery: the elimination of barrel Cheddar from the cheese price survey and the return to the *higher-of* class I skim milk pricing rule. The price calculations—*higher-of* mover and *blocks-only* cheese survey—operate on different aspects yet have complementary effects on base farm price. The *higher-of* aligns with the objective that class I generally remain the highest-valued use to encourage orderly marketing. Removing barrels reduces survey-driven noise on the manufacturing side, improving coherence of the cheese-protein signal. While this research focused on milk prices from a farm perspective, these changes also affect cooperatives, processors, and others in the dairy value chain. Market reactions and firm strategies will determine the ultimate effects of these pricing modifications in the long run.
